# The Asian tiger hunts in Maputo city—the first confirmed report of *Aedes* (*Stegomyia*) *albopictus* (Skuse, 1895) in Mozambique

**DOI:** 10.1186/s13071-016-1361-4

**Published:** 2016-02-09

**Authors:** Ayubo Kampango, Ana Paula Abílio

**Affiliations:** Laboratório de Entomologia, Instituto Nacional de Saúde (INS), Av. Eduardo Mondlane / Allende, nº 1008, C.P. 246, Maputo, Mozambique

**Keywords:** *Aedes albopictus*, New record, Dengue, Chikungunya, Maputo City, Mozambique

## Abstract

**Background:**

Increasing evidence suggests that dengue fever is endemic in Mozambique. Larvae of both the Afrotropical vector *Aedes aegypti* and its subspecies, *Ae. aegypti formosus,* have been reported from three provinces in Mozambique, two of which recently experienced dengue outbreaks. Despite reports of the invasive Oriental vector *Ae. albopictus* on the islands in the Mozambique Channel and nearby Indian Ocean, the species has not yet been reported in Mozambique.

**Findings:**

Four host-seeking female mosquitoes, collected biting the authors in an urban neighbourhood of Maputo City in the late afternoon of 6 December, 2015, are herein morphologically confirmed as *Ae. albopictus*.

**Conclusion:**

This is the first report confirming the occurrence in Mozambique of *Ae. albopictus*, an invasive species and an important vector of human arboviruses. In view of its potential role as a vector of dengue, Chikungunya and Zika viruses, studies are urgently needed to assess the geographical expansion and relative abundance of these important vectors to better understand the potential transmission impact of arboviruses that are efficiently transmitted and globally spread by these vectors.

## Background

Mozambique reported its first dengue (DEN) fever epidemics in 1984 and 1985 [1]. These were attributed to dengue virus serotype 3 (DENv-3), and evidenced the first DENv-3 transmission to man [1]. Following these outbreaks, the disease remained somewhat dormant until the first trimesters of 2014 and 2015, when two further dengue outbreaks occurred [2]. Recent detection of chikungunya virus (CHIKv) in acute febrile patients [3] strongly suggests that both DENv and CHIKv strains may be co-circulating in some northern and southern regions of the country, particularly in Pemba, and the capital city of Maputo [3].

The transmission of DEN and CHIK in Africa is attributed to the presence of the highly effective Afrotropical vector *Aedes* (*Stegomyia*) *aegypti* (Linnaeus, 1762) [4–6], which is abundant in urban and peri-urban areas throughout the tropics and subtropics [7, 8]. *Aedes aegypti* was first documented in Mozambique in 1960 [9] and since then, larvae of both *Ae. aegypti* and its subspecies *Ae.* (*Stg.*) *aegypti formosus* (Walker, 1848) have been reported from Maputo, Nampula and Cabo Delgado provinces [10]. The forest form *Ae. aegypti formosus* utilises tree holes and other natural larval sites and is considered predominantly zoophilic, whereas *Ae. aegypti* is highly adapted to man, breeding in small natural and artificial water collections in urban / semi-urban regions where it is highly anthropophilic [4, 11, 12]. The relative roles of these subspecies and other local potential vector taxa in dengue outbreaks in Mozambique are yet to be fully elucidated.

It is well documented that the leading Oriental arbovirus vector *Ae.* (*Stg.*) *albopictus* (Skuse, 1895), native to SE Asia and islands of the western Pacific and Indian Ocean [13, 14], is successfully invading tropical and temperate regions worldwide. Facilitated by its egg desiccation tolerance and bionomic plasticity [15], the species has successfully been introduced and become established globally by means of goods transportation, particularly through international trade of used tires [16, 17] and flowers [18]. These introductions are of particular concern as *Ae. albopictus* is an efficient vector of at least 26 viruses affecting man, including dengue, chikungunya, zika, yellow fever and Japanese encephalitis [19, 20], as well as some medically important filarial parasites, e.g. *Dirofilaria immitis* [21]. Nationwide arbosurveillance activities carried out between 1957 and 1959 did not detect the presence of *Ae. albopictus* in Mozambique [9]. However, the species has been found and incriminated as DEN and CHIK vectors on islands in the Mozambique Channel and nearby Indian Ocean [22, 23], and a recent modelling study indicated Mozambique as highly suitable for the establishment of *Ae. albopictus* populations [8].

## Findings

On the afternoon of December 6, 2015, the authors were gathered outdoors with three friends for a social gathering on the patio of an inhabited house in the densely inhabited Alto maé neighbourhood (25^o^57.548′S, 32^o^34.116′E) of Maputo, the capital city of Mozambique. Noting the unusually aggressive mosquitoes attacking their forearms and ankles, the authors collected the host-seeking females using hand-held aspirators. Four females collected between 14:00 and 16:00 were later tentatively identified as the exotic species, *Ae. albopictus.* Given this preliminary finding, further resting collections were undertaken in tyres and a discarded laundry tank around the property from 14:00 and 16:00 for the two following days (December 7 and 8, 2015), in an attempt to collect more specimens. Outdoor resting collections yielded an additional 21 samples, all later identified as *Culex* (*Culex*) *quinquefasciatus* Say 1823; no larval sites were detected in the surrounding gardens.

**Fig. 1:**
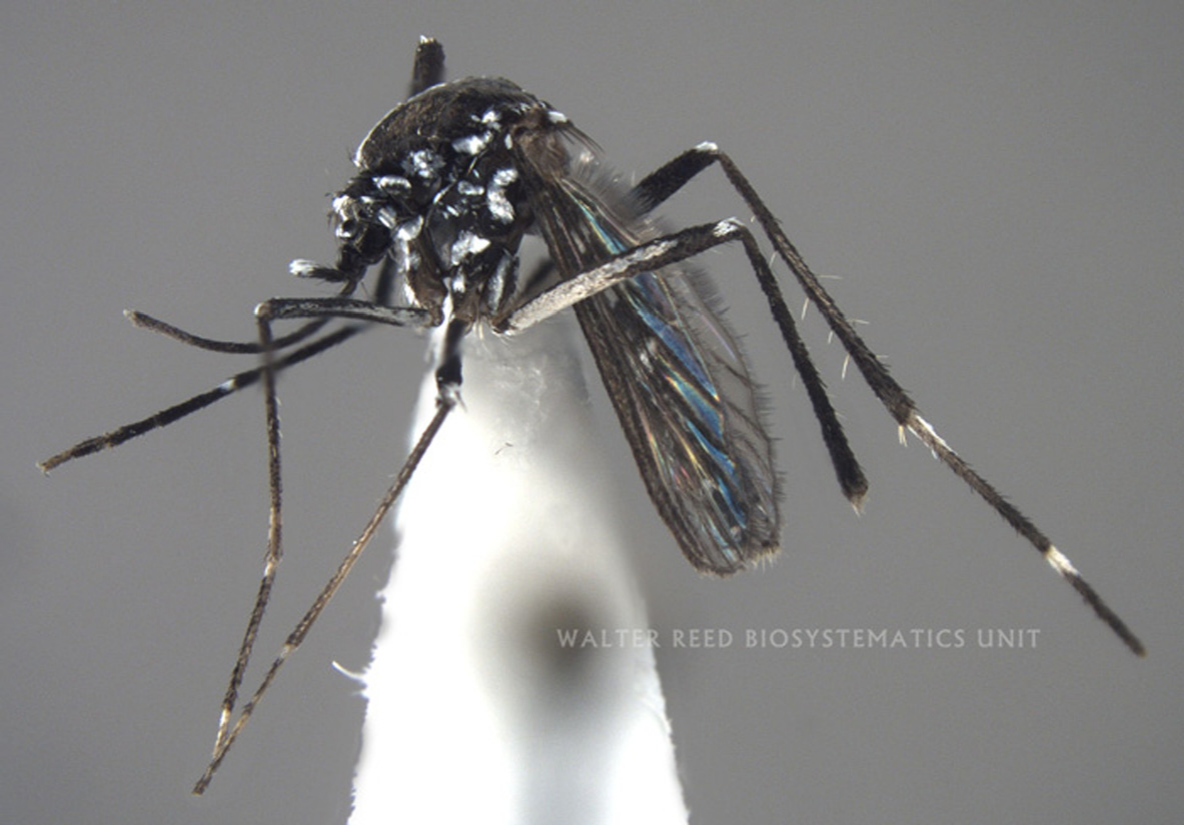
Habitas photograph of *Aedes albopictus* specimen MOZ 1. Coll: 6 December 2015, Alto maé neighbourhood (25^o^57.548’S, 32^o^34.116’E), Mozambique. Ayubo Kampango & Ana Paula Abílio, 14:00–16:00. Photograph by J. Stoffer (WRBU)

**Fig. 2:**
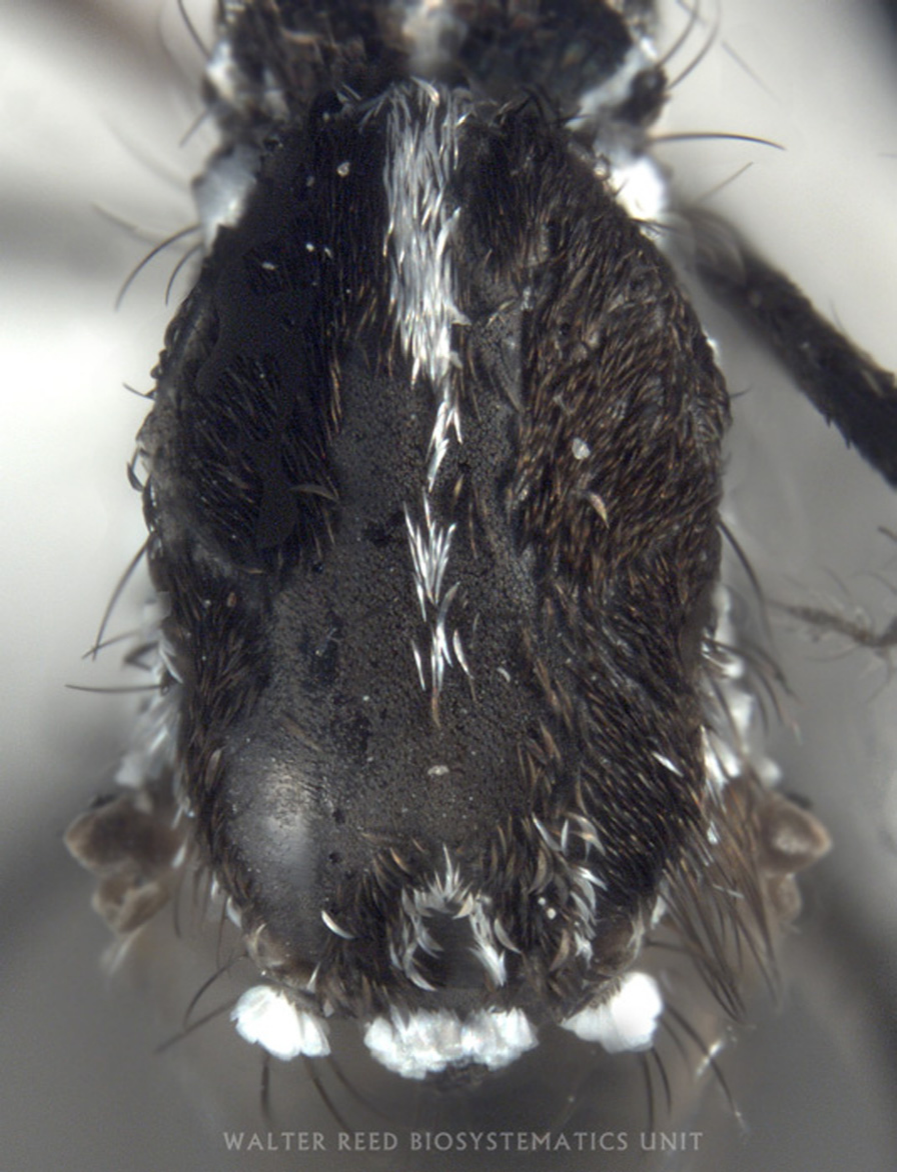
Dorsal view of thorax of *Aedes albopictus* specimen MOZ 1. Coll: 6 December 2015, Alto maé neighbourhood (25^o^57.548’S, 32^o^34.116’E), Mozambique. Ayubo Kampango & Ana Paula Abílio, 14:00–16:00. Photograph by J. Stoffer (WRBU)

**Fig. 3:**
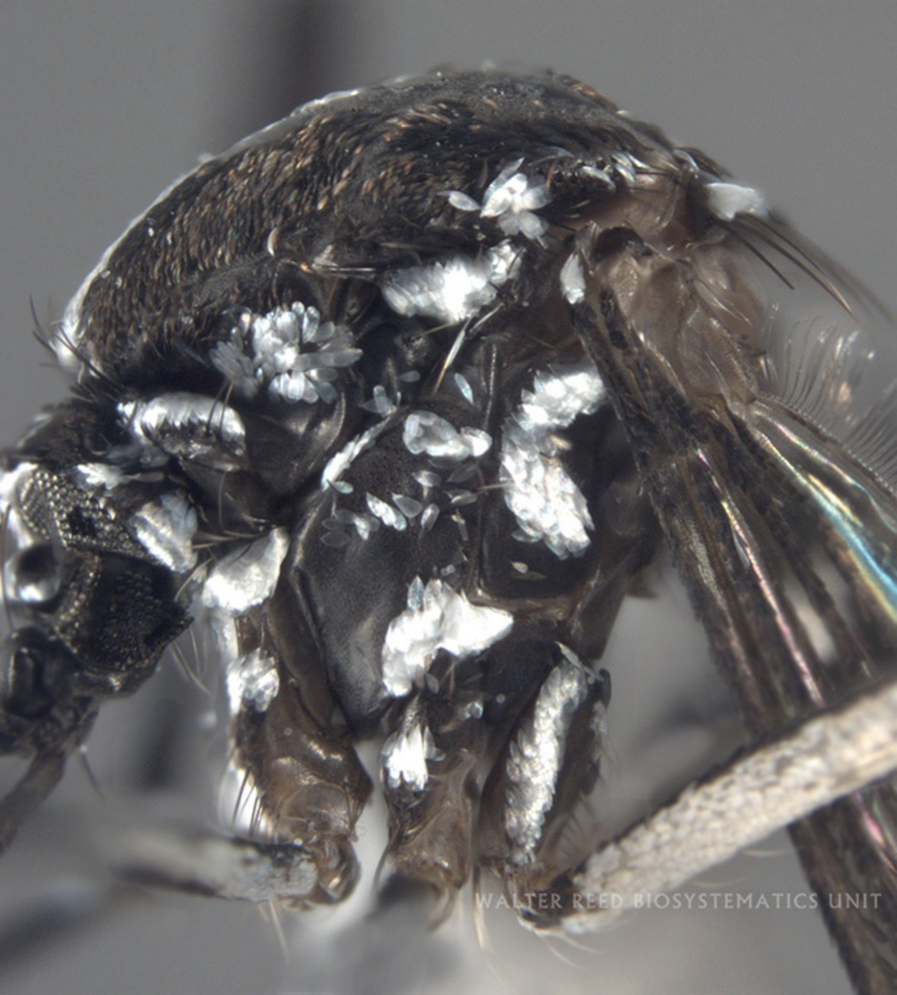
Lateral view of thorax of *Aedes albopictus* specimen MOZ 1. Coll: 6 December 2015, Alto maé neighbourhood (25^o^57.548’S, 32^o^34.116’E), Mozambique. Ayubo Kampango & Ana Paula Abílio, 14:00–16:00. Photograph by J. Stoffer (WRBU)

Suspected *Ae. albopictus* samples (*n* = 4) were taken to the Laboratory of Entomology, National Institute of Health (INS) in Maputo City for independent morphological identification by both authors using available taxonomic keys [11, 24, 25]. Specimens 1 (good specimen; see Fig. 1 a–c) and 3 were later sent to Dr Yiau-Min Huang (Walter Reed Biosystematics Unit, Maryland, USA) who verified the identifications, thus confirming the presence of *Ae. albopictus* in Mozambique for the first time. Voucher specimens of the four *Ae. albopictus* collected in this study are deposited in the insect repository of National Institute of Health (INS) in Maputo City, Mozambique. Specimens 1 and 3 are card-point mounted on insect pins, whereas specimens 2 & 4 are stored in individual Eppendorf® tubes. A full habitas photograph (Fig. 1) as well as dorsal (Fig. 2) and lateral views (Fig. 3) of the voucher specimen MOZ 1 are shown; equivalent photographs for voucher specimen MOZ 3 (not shown) are held by the authors.

Although some characters were rubbed in some specimens, the samples consistently exhibited the following unique morphological characters of *Ae. albopictus* as described by Huang [11, 24, 25] and listed (with permission) for clarity below: “…**Head**: Palpus approximately 0.2 length of proboscis, with white scales on apical half (Fig. 1). **Thorax:** Scutum with a prominent median stripe which narrows slightly posteriorly and forks at beginning of prescutellar space; a line of posterior dorso-central white scales on each side that does not reach to middle of scutum; a patch of broad, flat white scales on lateral margin just before level of wing root and few narrow curved white scales over wing root; posterior pronotum with a large patch of broad white scales; post-spiracular scales absent and subspiracular area with white scales; mesepimeral scale patches connected forming a V-shaped white scale patch (Figs. 2, 3). **Wing:** With dark scales on all veins except for minute basal spot of white scales on costa (Figs. 1, 3); first forked cell 1.5 times as long as its stem. **Abdomen:** Pale basal bands present on terga II-VII. **Legs:** Fore and mid-femur dark anteriorly and paler posteriorly; hind femur with a broad white stripe anteriorly which widens at base; fore- and mid- tarsi with basal white bands on tarsomeres l-2; hind tarsus with basal white bands on tarsomeres 1-4…”. The patch of broad flat white scales on lateral margin of the scutum just before level of wing root in *Ae. albopictus* is a critical character that separates this invasive species from other closely related Afrotropical taxa (Y.-M. Huang, pers. comm). These broad scales can clearly be seen in Fig. 3.

## Conclusion

This report comprises the first confirmed record of the SE Asian tropical invasive vector *Ae. albopictus* in the densely populated capital city of Matupo in Mozambique and is of high public health significance. Invasive populations of *Ae. albopictus* have been shown to utilise a diverse range of natural and artificial habitats in West Africa [26–29], and its introduction can greatly affect the distribution and the dynamics of native vectors populations, resulting a new patterns of disease transmission and risk profiles [30, 31]. Additional studies must be encouraged to thoroughly understand the local distribution, behaviour and vector competence of this invasive species, and its interactions with indigenous *Aedes* (*Stegomyia*) species, to better support future DEN and CHIK disease transmission disruption and effective vector control strategies in Mozambique.
